# Comparison of assessment techniques: plasma lipid and lipoproteins related to the metabolic syndrome

**DOI:** 10.1186/1476-511X-5-3

**Published:** 2006-01-31

**Authors:** Brenda M Davy, Kevin P Davy

**Affiliations:** 1 Dept of Human Nutrition, Foods and Exercise (0430), Virginia Tech, Blacksburg, VA 24061, USA

## Abstract

**Background:**

The purpose of this investigation was to determine the influence of analytical method on reported concentrations of plasma lipids and lipoproteins, and to determine if there are clinical implications of any potential differences on identification of the metabolic syndrome dyslipidemia, CVD risk stratification and classification of LDL subclass phenotype.

**Results:**

Plasma triglyceride (TG) concentrations were 1.09 ± 0.06 and 1.17 ± 0.06 mmol/L and plasma high density lipoprotein cholesterol (HDL-C) concentrations were 1.09 ± 0.03 vs 1.19 ± 0.03 mmol/L (both p < 0.05) from 113 duplicate samples sent to two laboratories utilizing different lipid and lipoprotein analytical methods (LABS 1 and 2, respectively). Plasma total cholesterol and low-density lipoprotein cholesterol (LDL-C) concentrations were also significantly different between laboratories. Spearman rho correlations indicate excellent agreement of TG and HDL-C determined by the two laboratories (r = 0.96, TG; r = 0.91, HDL-C, both p < 0.001). Eleven vs. 14 individuals met the TG criteria and 70 vs. 48 met HDL-C metabolic syndrome criteria with LAB 1 and 2, respectively. Apoprotein B concentration (LAB 1) and LDL particle number (LAB 2) were highly correlated. (r = 0.92, P < 0.01). LAB 2 characterized more individuals as LDL pattern B phenotype, as compared to LAB 1 (30 vs. 14%, P < 0.05).

**Conclusion:**

Different plasma lipid and lipoprotein analytical techniques yield results which are highly correlated, yet significantly different, which suggests a consistent measurement difference. This difference has clinical implications, in that the proportion of individuals identified as meeting the metabolic syndrome dyslipidemia criteria, "at risk" based upon apo B or LDL particle number, and the LDL pattern B phenotype will differ based upon choice of analytical method.

## Background

The metabolic syndrome is a condition in which several abnormalities (hypertension, insulin resistance, dyslipidemia, and impaired fibrinolysis) are clustered together with elevated abdominal visceral fat as the central feature. Individuals with the metabolic syndrome, estimated to be approximately 24% of US adults, are at increased risk for developing diabetes and cardiovascular disease (CVD)[1-3]. One objective of the clinical management of the metabolic syndrome is the treatment of dyslipidemia, which is characterized by blood triglyceride (TG) concentrations above 1.69 mmol/L (150 mg/dl) and high-density lipoprotein cholesterol (HDL-C) concentrations below 1.03 mmol/L (40 mg/dl) in men and 1.29 mmol/L (50 mg/dl) in women [4].

The predominance of small, dense low-density lipoprotein (LDL) particles, termed LDL subclass pattern B phenotype, and apoprotein (apo) B concentration, which reflect the number of circulating atherogenic lipoprotein particles, have also been identified as important clinical markers of CVD risk [5,6]. Importantly, the pattern B phenotype is associated with the metabolic syndrome [7]. Small, dense LDL particles are cholesterol-depleted; therefore the use of total cholesterol (TC) and LDL-C concentrations may not reflect the number of circulating atherogenic particles in the plasma [8]. For this reason, concentration of apo B (or LDL particle number, a closely related variable) may be useful in identifying and monitoring patients at risk for CVD [8]. Furthermore, it has been suggested that apo B concentrations be used in place of traditional lipid parameters (TC, LDL-C, HDL-C, and triglycerides) to assess and monitor CVD risk [8-10]. Individuals with both the pattern B phenotype and a high apo B concentration have a six-fold increased risk of ischemic heart disease compared to those without these characteristics [5].

Various analytical methods are available for the determination of plasma lipid and lipoprotein concentrations and characteristics. Traditional methods include enzymatic method determination of TC, TG, and HDL-C and gradient gel electrophoresis (GGE)(LAB 1) for assessment of lipoprotein particle size and subclasses, while newer technology utilizes nuclear magnetic resonance (NMR) spectroscopy (LAB 2) for assessment of these variables [11]. Both laboratories report similar coefficient of variations (CVs) of ~1–3% for lipid concentrations (TC, TG, HDL), while reported CVs for apo B (LAB 1) and LDL particle number (LAB 2) are ~6% and 2%, respectively [11,12]. An advantage of the technique employed by LAB 1 is that it has been in use for longer period of time and is thus widely accepted, while an advantage of the newer NMR technique is that it is much less time- and labor-intensive. Other potential differences between analytical techniques include sample volume and cost. Analytical method may influence plasma lipid and lipoprotein concentrations and characteristics, and it is not clear if these potential differences have clinical implications (ie, identification of metabolic syndrome dyslipidemia or CVD risk stratification). Therefore, the purpose of this investigation was twofold: 1) to determine the influence of analytical method (LAB 1 vs. LAB 2) on reported concentrations of plasma lipids and lipoproteins, 2) to determine if there are clinical implications for these differences (ie, on clinical identification of the metabolic syndrome dyslipidemia, CVD risk stratification and classification of LDL subclass phenotype).

## Results and Discussion

As shown in Table 1, TG and HDL-C concentrations were significantly higher (mean difference = 0.08 mmol/L and 0.10 mmol/L for TG and HDL-C, respectively), while total cholesterol and LDL-C concentrations were significantly lower according to LAB 2. Consequently, clinicians utilizing LAB 1 with these results would be less likely to categorize individuals as meeting the ATP III criteria for TG concentration, but more likely to categorize individuals as meeting the ATP III criteria for HDL-C concentration. In spite of these significant differences, the proportion of individuals categorized as meeting both metabolic syndrome dyslipidemia criteria were similar (ie, 11 vs. 9 individuals from LABS 1 and 2, respectively). In addition, the measurement differences between laboratories were consistent as determined by the high rank order correlation of variables (Table 1).

**Table 1:** Plasma Lipid and Lipoprotein Concentrations from Laboratories Utilizing Different Analytical Methods

**Variable**	**LAB 1**	**LAB 2**	**Correlation^B^**
Total Cholesterol, mmol/L	4.13 ± 0.08	3.82 ± 0.08^A^	0.92^C^
HDL-C, mmol/L	1.09 ± 0.03	1.19 ± 0.03^A^	0.91^C^
LDL-C, mmol/L*	2.53 ± 0.08	2.25 ± 0.05^A^	0.88^C^
Triglycerides, mmol/L	1.09 ± 0.06	1.17 ± 0.06^A^	0.96^C^
Number in sample meeting ATP III criteria			
Triglycerides, n (%)	11 (10%)	14 (12%)^D^	0.78^C^
HDL-C, n (%)	70 (61%)	48 (42%)^AD^	0.60^C^

*calculated, Friedewald equation.

^A^P < 0.05 vs. LAB 1.

^B^Spearman's rho.

^C^Significant correlation (P < 0.01).

^D^Significant difference, Chi square (Trig: χ^2 ^= 69.2, HDL-C: χ^2 ^= 40.6; both P < 0.0001).

**Figure 1:**
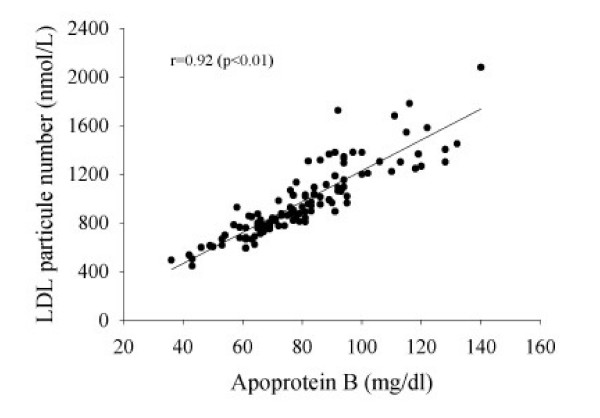
Relationship of Apoprotein B Concentration (LAB 1) to LDL Particle Number (LAB 2).

With respect to the number of circulating atherogenic particles in the plasma, the two laboratories differ slightly in the type of information provided. LAB 1 measures apo B concentration, and LAB 2 measures LDL particle number. The two variables are not identical but should be correlated, as each LDL particle carries one apoprotein B moiety. However, apo B is also carried on very-low density lipoproteins and intermediate density lipoproteins. These findings (see Figure 1) indicate that the two measures are highly correlated (r = 0.92, P < 0.01), even when analyzed at different laboratories. Since it has been suggested that apo B (or LDL particle number) be used in place of traditional lipid parameters (ie. TC, LDL-C, HDL-C, and triglycerides) to assess CVD risk [8-10], the number of individuals in our sample at increased risk according to apo B concentrations (>120 mg/dl; 13) measured by LAB 1 as compared to LDL particle number (1300–1599 nmol/L; 14) measured by LAB 2 were compared. Using laboratory-specific criteria, six individuals were identified as being at increased risk based upon apo B concentrations (LAB 1), while 20 individuals at increased risk based upon LDL particle number (LAB 2). Mean 10-year Framingham risk scores [4] for these individuals identified at increased risk based upon apo B or LDL particle number were 3.8% (± 1.5) and 1.5% (± 0.5) at LABS 1 and 2, respectively. The latter suggests that clinicians utilizing LAB 1 would treat fewer individuals at higher CVD risk relative to LAB 1 if apo B was used in place of traditional lipid parameters, as the Framingham risk score utilizes TC and HDL-C concentrations. Conversely, utilizing LAB 2 would identify more individuals for intervention based upon LDL particle number; those advocating for a more aggressive approach for reduction of lipids and lipoproteins may view this as advantageous. Direct comparison of the predictive value of LDL particle number vs. apo B concentration to CVD risk [15] and correlation with metabolic syndrome features [15] suggest that LDL particle number may be superior.

**Figure 2:**
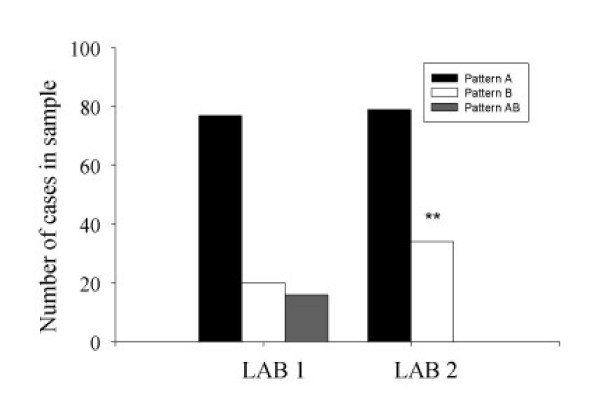
LDL Phenotype Classification According to Laboratories Utilizing Different Analytical Methods*. *Lab 1: Pattern B = LDL particle size < 22.0–25.74 nm; AB = 25.75–26.34 nm; Pattern A = 26.3–28.5 nm. Lab 2: Pattern B = LDL particle size ≤20.5 nm; Pattern A = 20.6–22.0 nm. **Significant difference from LAB 1; Χ^2 ^= 64.6, P < 0.0001.

High correlations (r = 0.89) have been reported between LDL particle sizes determined by GGE and NMR [15]. However, despite high correlations, differences may still exist in categorization of individuals according to LDL phenotype based upon LDL particle size measurement when determined by different analytical methods. In our comparison, LAB 2 was more likely to categorize individuals as having the pattern B phenotype (see Figure 2); this may in part be due to the use of the intermediate (AB) phenotype classification by LAB 1 but not LAB 2. This may have clinical significance in that an individual identified as pattern B is at increased risk for CVD [5] and should be evaluated for other features of the metabolic syndrome [7]. Furthermore, the therapeutic treatment approach may vary according to LDL phenotype classification [17], which could be problematic if an individual is misclassified. Thus, follow-up testing of LDL particle size after implementation of treatment would seem prudent to ensure the appropriate treatment response.

## Conclusion

Various laboratories offer plasma lipid and lipoprotein assessment beyond the traditional lipid and lipoprotein panel, such as lipoprotein characteristics (eg, particle size) and number. These findings indicate that results may vary according to the analytical method used, and that this variability may have clinical implications for assessment of CVD risk. Results from the two laboratories studied in this investigation were significantly different; however, the measurement difference was consistent (eg, if an individual's reported TG value was elevated according to LAB 1, it was likely to be elevated according to LAB 2).

Individuals with the metabolic syndrome dyslipidemia risk determinants and with LDL subclass pattern B phenotype should be identified clinically, as they are at increased risk for CVD. However, the laboratory method used to analyze lipid and lipoprotein variables for CVD risk stratification and diagnosis of the metabolic syndrome dyslipidemia appears to impact the proportion of individuals identified to be "at-risk". Results from LAB 2 would categorize more individuals with the LDL pattern B phenotype, as well as at increased CVD risk based upon LDL particle number. Borderline results may warrant follow-up testing to ensure the appropriateness of risk categorization as well as the treatment approach employed.

Thus, lipid and lipoprotein results from the two laboratories studied were highly correlated (ie, r = 0.88–0.96), but differ significantly in their categorization of individuals at risk for CVD. These differences may lead to different clinical treatment strategies.

## Methods

Duplicate fasting plasma samples were obtained from 113 (70 men, 43 women) participants of a cross-sectional research study. Participants were heterogeneous in terms of age (range 18–70 years), activity level (V0_2 _max range 19.7–82.0 ml/kg/min), and body weight (BMI range 17.8–39.9). Participants were excluded if they had a history of cardiovascular disease, diabetes, or other major chronic disease. Physical characteristics (mean ± SEM) of participants were as follows: age 31.1 (± 1.2) years; height 173.6 (± 1.0) cm; weight 79.1 (± 1.4) kg; BMI 26.2 (± 0.4) kg/m^2^. All participants provided informed consent prior to their participation in the investigation.

Plasma samples were sent to two commercial laboratories specializing in plasma lipid and lipoprotein analyses. Both laboratories are CLIA-certified (Centers for Medicare and Medicaid Services Clinical Laboratory Improvement Amendments). One laboratory (LAB 1) utilizes enzymatic methods (TG, HDL-C, total cholesterol) plus gradient gel electrophoresis (particle size, subclasses) [13,18] and the other laboratory (LAB 2) utilizes nuclear magnetic resonance spectroscopy [11]. With regard to analysis of the number of circulating atherogenic particles, LAB 1 reports apo B concentrations (mg/dl), while LAB 2 reports LDL particle number (nmol/L). Concentrations of total cholesterol, TG, HDL-C, LDL-C, LDL particle number and apo B, and LDL subclass phenotype classification (Pattern A/B) were compared between laboratories. In addition, the proportion of individuals meeting ATP III dyslipidemia criteria according to results reported by the two laboratories was compared.

Spearman's rho rank order correlational analysis was used to assess agreement in ranking of variables between LABS 1 and 2, and paired samples t-tests were used to determine differences between laboratories. Chi square test of independence analysis was used to determine if the number of individuals classified as LDL pattern B phenotype and those meeting ATP III criteria for TG and HDL-C differed between laboratories (SPSS v. 12.0). Significance was determined as P < 0.05. All data are expressed as Mean ± SEM.

## Authors' contributions

Brenda M. Davy: experimental design, data analysis and interpretation, manuscript preparation

Kevin P. Davy: data generation, editing, manuscript approval
